# Lack of Developmental Redundancy between Unc45 Proteins in Zebrafish Muscle Development

**DOI:** 10.1371/journal.pone.0048861

**Published:** 2012-11-07

**Authors:** Sophie A. Comyn, David Pilgrim

**Affiliations:** Department of Biological Sciences, University of Alberta, Edmonton, Alberta, Canada; Fred Hutchinson Cancer Research Center, United States of America

## Abstract

Since the majority of protein-coding genes in vertebrates have intra-genomic homologues, it has been difficult to eliminate the potential of functional redundancy from analyses of mutant phenotypes, whether produced by genetic lesion or transient knockdown. Further complicating these analyses, not all gene products have activities that can be assayed *in vitro*, where the efficiency of the various family members can be compared against constant substrates. Two vertebrate UNC-45 homologues, *unc45a* and *unc45b*, affect distinct stages of muscle differentiation when knocked down in cell culture and are functionally redundant *in vitro*. UNC-45 proteins are members of the UCS (UNC-45/CRO1/She4p) protein family that has been shown to regulate myosin-dependent functions from fungi to vertebrates through direct interaction with the myosin motor domain. To test whether the same functional relationship exists between these *unc45* paralogs *in vivo*, we examined the developmental phenotypes of doubly homozygous *unc45b^−/−^*; *unc45a^−/−^* mutant zebrafish embryos. We focused specifically on the combined effects on morphology and gene expression resulting from the zygotic lack of both paralogs. We found that *unc45b^−/−^* and *unc45b^−/−^*; *unc45a^−/−^* embryos were phenotypically indistinguishable with both mutants displaying identical cardiac, skeletal muscle, and jaw defects. We also found no evidence to support a role for zygotic Unc45a function in myoblast differentiation. In contrast to previous *in vitro* work, this rules out a model of functional redundancy between Unc45a and Unc45b *in vivo*. Instead, our phylogenetic and phenotypic analyses provide evidence for the role of functional divergence in the evolution of the UCS protein family.

## Introduction

Members of the UCS (**U**NC-45/**C**RO1/**S**he4p) protein family are present in single-celled eukaryotes and metazoa [1] and participate in essential myosin-dependent functions by directly interacting with the myosin motor domain. This interaction is mediated by a conserved 400 residue carboxyl-terminal domain, a defining feature of UCS proteins [1], [2]. In contrast to the fungal UCS homologues, metazoan UNC-45 proteins also contain an amino terminal tetratricopeptide repeat (TPR) domain which facilitates the interaction between UNC-45 and Hsp90 [3], [4]. A flexible central region then joins the TPR and UCS domains and acts in concert with the UCS to bind to, and mediate the folding of, the myosin head domain [3], [4]. Through the formation of a stoichiometric complex with Hsp90, UNC-45 acts *in vitro* as both a direct myosin chaperone and an Hsp90 co-chaperone [4].

UNC-45 is essential for muscle assembly and function in *C. elegans* where it co-localizes with thick filaments [3], [5], [6], [7]. Thick filaments in *C. elegans* are assembled from two different myosin heavy chains, MHC A and B, which are asymmetrically arranged. The minor myosin isoform (MHC A) is found only in the central 2 µm of the filament, while MHC B is found along the majority of the lateral arms [8]. UNC-45 shows myosin isotype specificity, interacting with muscle myosin heavy-chain B (MHC B) through the UCS domain, but not with MHC A [1], [3], [6], [9]. If MHC B is removed (as in *unc-54* null mutants), *C. elegans* muscle thick filaments can be induced to form using only MHC A [10]. In such animals UNC-45 no longer localizes to the thick filament, and missense mutations in *unc-45* have no effect on thick filament assembly [6]. Thus, MHC A does not require UNC-45 activity despite the fact that the two myosins are 65% identical in sequence, and MHC A can functionally substitute for MHC B [11].

Vertebrates have two UNC-45 genes, *unc45a* and *unc45b*, whose expression patterns differ dramatically [12], [13], [14]. Unc45b expression is restricted to striated muscle, where it interacts with Hsp90 during myofibrillogenesis and is required for folding the myosin motor domain [4], [13]. In humans, Unc45b is a candidate locus for cardiomyopathies and a protein determining hereditary inclusion-body myopathy (p97) regulates Unc45b stability [15], [16]. Knockdown of *unc45b* and *hsp90a* in zebrafish or *Xenopus* produce similar phenotypes, including cardiac dysfunction, disordered sarcomeres and paralysis, and regulation of these two genes is interdependent [13], [17]. Genetic mutations affecting *unc45b* have been identified in zebrafish (*steif*) and *Xenopus* (*dicky ticker*) [13], [14], [18]. The *unc45b* mutant *steif* has defective myofibrils containing disorganized thick and thin filaments and reduced levels of muscle myosin protein [13], [14]. Consequently, embryos are paralyzed, lack circulation, and develop extensive heart and yolk edema by 5 days post-fertilization (dpf). As is seen in *C. elegans* for *unc-45*, overexpression of *unc45b* in zebrafish leads to severely disorganized myosin filaments, indicating the need for tight regulation of unc45b expression [19].

Both Unc45a and Unc45b are expressed in differentiating C2C12 myoblasts and such cells show defects in myogenesis when expression is reduced [12]. Antisense-oligonucleotides directed against Unc45a affected myoblast proliferation, while Unc45b knockdown affected sarcomere formation [12]. *In vitro*, both Unc45a and Unc45b have the capacity to fold the myosin motor domain in an Hsp90/ATP-dependent manner [20]. In that assay, Unc45a was shown to be the better UNC-45 chaperone for myosin folding [20]. In zebrafish and mouse, *unc45a* has a broad expression pattern with transcripts present in the brain, pharyngeal arches, and retina, although no significant expression is seen in the striated muscles of the trunk or heart [12], [21]. Unexpectedly, a mutation in zebrafish *unc45a (kurzschluss)* results in aortic arch defects, which is not easily explained via a role in myosin regulation [21]. The *unc45a* mutants develop an arteriovenous malformation involving aortic arches 5 and 6 that causes blood to shunt from the primary head sinus back into the heart. In contrast to *unc45b*, myofibril organization and thick filament assembly appear normal in *unc45a* mutants. These observations have led to the hypothesis that Unc45a may either regulate myosin assembly in non-muscle cells, or contribute to muscle myosin assembly solely in muscle groups other than the trunk or heart.

In *C. elegans*, the single *unc-45* gene is responsible for both striated muscle assembly and function through the muscle myosin UNC-54/MHC-B, as well as cytokinesis and cell polarity via the type II non-muscle myosin NMY-2 [6], [22], [23]. These observations, in conjunction with those from the UCS protein family member Rng3, which also interacts with a type II myosin and is required for cytokinesis [24], led to hypotheses that the vertebrate orthologues of UNC-45 may divide these functions between them. A cytokinesis defect has not been reported for either of the *unc45* mutants in zebrafish [14], [21]. Several models could explain both the *in vitro* biochemical redundancy toward myosin folding and the *unc45a* aortic arch phenotype. First, functional redundancy between the two vertebrate Unc45 proteins could be masking a cytokinesis phenotype in either of the *unc45* zebrafish mutants. Second, Unc45a contributes to muscle myosin assembly only in the tissues in which it is expressed and only during later stages of development. A subset of specific muscles lacking both isoforms would express a phenotype more severe than that seen in the single *unc45b* mutant. Finally, given the striking lack of a muscle phenotype in the zebrafish *unc45a* mutant, Unc45a function is not required for myogenesis in zebrafish, but is required in mouse C2C12 cells. The availability of putative null mutants in both zebrafish homologues provides us a unique opportunity to test these hypotheses and examine developmental redundancy between two members of a highly conserved protein family.

Here we describe the phenotypic characterization of doubly mutant *unc45b^−/−^; unc45a^−/−^* zebrafish embryos with an emphasis on the combined effects resulting from the loss of both paralogs. Of special interest were regions where the expression domains of *unc45a* and *unc45b* may overlap, such as in the pharyngeal arches at later stages of development. Our results effectively rule out any functional redundancy between Unc45a and Unc45b *in vivo* and provide evidence for the role of functional divergence between the UNC-45 proteins during vertebrate evolution.

## Methods

### Ethics Statement

This study was carried out in strict accordance with the recommendations from the Canadian Council for Animal Care. The protocol was approved by the Animal Care and Use Committee of the University of Alberta.

### Fish lines, maintenance and genotyping

Zebrafish were maintained according to standard procedures [25]. Adults were naturally spawned to obtain embryos, which were raised at 28.5°C and staged according to published morphological hallmarks [26]. Embryos analyzed past the 24 hpf stage were incubated in embryo medium supplemented with 0.003% phenylthiourea to prevent melanin formation [25]. All procedures were carried out in compliance with the guidelines stipulated by the Canadian Council for Animal Care and the University of Alberta. The *steif*/*unc45b* ^+/sb60^ and *kus*/*unc45a* ^+/tr12^ strains have been previously described and were generously shared with us [13], [21]. Homozygous *steif*/*unc45b* ^+/sb60^, homozygous *kus*/*unc45a* ^+/tr12^, and the trans-heterozygous *unc45b* ^+/sb60^ *; unc45a* ^+/tr12^ animals were used for all experiments. Genotypes were determined using derived cleaved amplified polymorphic sequence (dCAPS) analysis for all experiments that required the identification of either homozygous or heterozygous double mutants, or was conducted at a developmental stage prior to when mutants could be scored phenotypically [27], [28]. Throughout the text, we abbreviate the genotypes as: *unc45a* ^−/−^, *unc45b* ^−/−^, and *unc45b* ^−/−^ *; unc45a* ^−/−^ to refer to single and double mutants of the alleles above.

### Phylogenetic analysis

Phylogenetic analysis was performed using the MEGA5 program [29]. Amino acid sequences were retrieved from Ensembl (http://www.ensembl.org/index.html) and aligned using ClustalW2 [30]. Phylogenetic trees were generated by the neighbour-joining method using default parameters and 1,000 bootstrap replicates. Ensembl identification numbers for all sequences used in analyses are found in Table S1.

### mRNA *in situ* hybridization and detection

Antisense RNA probes were synthesized by *in vitro* transcription using T7 RNA polymerase (Ambion) and digoxigenin-11-UTP RNA labeling (Roche). Probes (800–1,000 bases) were made from PCR template amplified using gene-specific primers. The 5′ ends of the reverse primers incorporated an RNA polymerase promoter sequence (T3 or T7) to enable RNA production [31]. The unc45a primers create a probe that is 816 bp in length that spans the unc45a mRNA sequence from bases 26–841. When aligned to the unc45b mRNA sequence this corresponds to bases 2–787. There are 389 bases shared between the *unc45a* probe and the *unc45b* mRNA sequence (the largest span of identity is 11 bases).

Whole-mount *in situ* hybridization analysis was performed essentially as described [32] with the following modifications. Dechorionated embryos were fixed overnight at 4°C and then stored in 100% methanol at −20°C. Prior to resuming the protocol, embryos were rehydrated in a methanol/PBS-Tw series (PBS with 0.1% Tween-20). Embryos were permeabilized with 10 µg/mL Proteinase K in PBS-Tw. Permeation times depended on embryo age: 27 hpf, 3 minutes; 48 hpf, 20 minutes; 3 dpf, 25 minutes; 4 dpf, 35 minutes. Embryos were imaged using an Olympus stereoscope with a QImaging Micropublisher camera. Figures were assembled using Adobe Photoshop CS Version 8.0.

### Immunohistochemistry and histology

Embryos were fixed in 2% TCA and washed four times with 0.8% Triton X-100 in PBS (PBS-Tx) for 5 minutes at room temperature. Samples were blocked in 5% BSA in PBS-Tx for 1 hour at room temperature and incubated overnight at 4°C in a 1∶10 dilution of monoclonal antibody MF-20 (Developmental Studies Hybridoma Bank) in blocking solution. Embryos were washed five times with PBS-Tx for 5 minutes at room temperature. Subsequent to a single 5 minute PBS wash, embryos were incubated overnight at 4°C in a 1∶1,000 dilution of anti-mouse Alexa 488 (Molecular Probes) secondary antibody. Antibody was removed by three washes in PBS-Tx for 10 minutes each at room temperature prior to mounting embryos in 3% methylcellulose. Images were captured using a Nikon Eclipse 80i confocal.

Alcian Blue stains proteoglycan components of the extracellular matrix [33], [34]. The following method is adapted from [35]. 5 dpf larvae were fixed 2 hr in 4% PFA and then dehydrated in 50% ethanol for 10 minutes. Once dehydrated, larvae were stained overnight in: 0.02% Alcian Blue (Sigma), 60 mM MgCl_2_, and 70% ethanol. Following a rinse in water, embryos were bleached for 20 minutes in equal volumes of 3% H_2_O_2_ and 2% KOH in open tubes on the bench top. To visualize cartilage more readily, tissues were cleared for 20 minutes with gentle rocking using 1 mg/mL trypsin dissolved in saturated sodium tetraborate [36]. Larvae were cleared in 20% glycerol with 0.25% KOH for 1 hour followed by 50% glycerol with 0.25% KOH for 2 hours and stored at 4°C in 100% glycerol.

## Results

### Phylogenetic analysis of UNC-45

**Figure 1:**
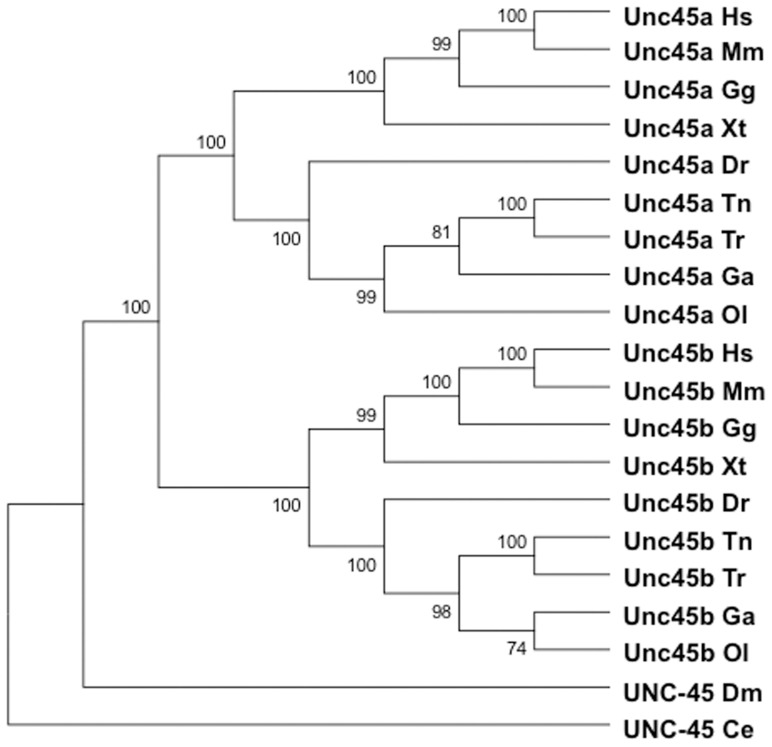
Phylogenetic analysis of Unc45 sequences. Protein sequences were aligned using ClustalW2 and phylogenetic trees were generated by the Neighbour-Joining method with MEGA5. Bootstrap values for 1000 replicates are indicated to the right of the nodes. Ce, *Caenorhabditis elegans*; Dm, *Drosophila melanogaster*; Dr, *Danio rerio*; Ga, *Gasterosteus aculeatus*; Gg, *Gallus gallus*; Hs, *Homo sapiens*; Mm, *Mus musculus*; Ol, *Oryzias latipes*; Tn, *Tetradon nigroviridis*; Tr, *Takifugu rubripes*; Xt, *Xenopus tropicalis*. The Ensembl gene IDs of the genes used in generating the phylogenetic trees are listed in Table S1.

Although it has been suggested that the UNC-45 gene duplication in vertebrates emerged during the chordate radiation, it is unclear whether the two UNC-45 genes present in all vertebrates share a common origin dating to early in the chordate lineage, or whether those present in teleosts are unique, having arisen from a separate whole-genome duplication event in the common ancestor to teleosts followed by selective loss [12], [37]. To clarify the evolutionary relationship between UNC-45 proteins we performed a phylogenetic analysis before embarking on a detailed phenotypic analysis of the *unc45b^−/−^; unc45a^−/−^* mutant (Fig. 1). In particular, we tested whether sequence divergence patterns could help to clarify the phylogenetic relationship between the two vertebrate UNC-45 genes.

A neighbour-joining tree generates three major sequence groups: Unc45a, Unc45b, and UNC-45 (Figure 1). Vertebrate genomes contain two UNC-45 gene copies, whereas non-vertebrates have a single UNC-45 gene. In vertebrates, both Unc45a and Unc45b amino acid sequences share more similarity with homologs in other vertebrate species than with each other. For example, the *Danio rerio* Unc45a and Unc45b proteins are 77% similar to each other, but are ∼85% similar to human Unc45a and Unc45b, respectively. The UNC-45 amino acid sequence from *Drosophila melanogaster*, and Unc45a and Unc45b from *Danio rerio* are all approximately 60% similar to the single *Caenorhabditis elegans* UNC-45 protein.

One fifth of all *D. rerio* genes have been retained as duplicates following the whole-genome duplication event thought to have occurred prior to the teleost radiation, approximately 350 million years ago [38], [39]. We found no evidence for additional copies of either Unc45a or Unc45b in our searches of *T. rubripes*, *O. latipes*, *G. aculeatus*, *T. nigroviridis*, or *D. rerio* genomes, suggesting that the Unc45 gene duplicates, if generated at that time, were lost early during the teleost lineage. Therefore, it appears that vertebrate Unc45a and Unc45b have diverged equally from a single ancestral UNC-45 locus and given the comparable sequence divergence from their human orthologs, it is likely that the two Unc45 proteins were generated early in the vertebrate lineage following a whole-genome duplication event.

### Gross morphology of *unc45* mutants

We used a genetic approach to examine the functional relationship between *unc45* genes *in vivo*. The phenotypic assessment of *unc45b^−/−^; unc45a^−/−^* mutants focused on gross morphology and gene expression. Novel phenotypes not present in the single mutants, or a double mutant displaying phenotypes more severe than those present in the single mutants, would be consistent with functional redundancy between the two vertebrate *unc45* genes. The *unc45b^−/−^; unc45a^−/−^* mutant line was created through the crossing of adult *unc45b* ^+/−^ and *unc45a* ^+/−^ fish that have been previously described [13], [21]. The two mutants are predicted to be molecular nulls, since each is a nonsense mutation in the UCS domain, and in each case, morpholino-oligonucleotide knockdown of the cognate gene in wild-type embryos phenocopies the mutant phenotype [13], [14], [21]. Since progeny that are homozygous at either of the *unc45* loci are zygotic-lethal, strains were maintained as *unc45b* ^+/−^; *unc45a* ^+/−^ double heterozygotes. Crosses between these fish yielded embryonic progeny genotypes in the expected Mendelian ratios (data not shown). Specifically, no underrepresentation of the *unc45b^−/−^; unc45a^−/−^* genotype was observed, which might have suggested lethality during early developmental stages. Additionally, no evidence for a heterozygous effect was observed such that *unc45* ^+/−^ and *unc45* ^+/+^ genotypes appeared phenotypically identical in all combinations.

In regard to gross morphology, the *unc45b^−/−^*; *unc45a^−/−^* and *unc45b^−/−^* embryos appear identical (Fig. S1.). Both mutants are paralyzed and *unc45b^−/−^*; *unc45a^−/−^* embryos have reduced somite birefringency as has been reported previously for the *unc45b^−/−^* mutants [13], [14]. The absence of both circulation and cardiac contraction in the *unc45b^−/−^* and *unc45b^−/−^*; *unc45a^−/−^* mutants, results in accumulation of fluid in the pericardial space (cardiac edema) and yolk sac edema by 5 dpf. To a lesser extent, the *unc45a^−/−^* mutants also develop cardiac edema, but yolk sac edema does not advance to the degree seen in the other mutants.

### Differential distribution of *unc45a* and *unc45b* transcripts

**Figure 2:**
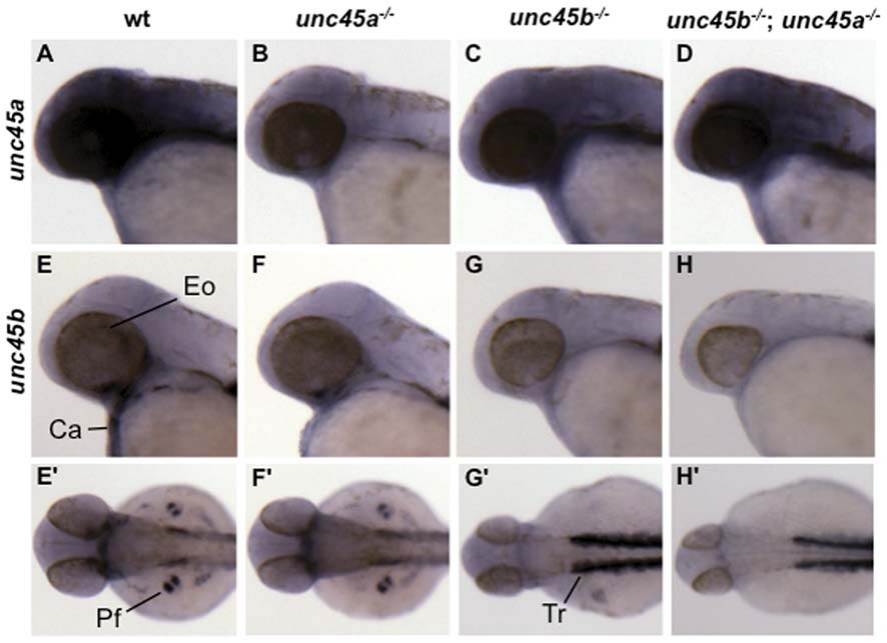
Comparison of *unc45a* and *unc45b* mRNA expression at 48 hpf. Wild type siblings (a,e,e'); *unc45a^−/−^* (b,f,f'); *unc45b^−/−^* (c,g,g'); and *unc45b^−/−^*; *unc45a^−/−^* (d,h,h') mutants. *unc45a* expression is diffuse in the region of the brain and pharyngeal arches. *unc45b* mRNA expression from lateral (e–h) and dorsal (e'–h') views, head to the left. Wild type siblings (e,e') and *unc45a^−/−^* (f,f') mutants express *unc45b* in the extraocular (Eo), cardiac (Ca), trunk (Tr), and pectoral fin (Pf) muscles. Expression is either minimal or absent in the extraocular, cardiac, and pectoral fin muscles of the *unc45b^−/−^* (g,g') and *unc45b^−/−^*; *unc45a^−/−^* (h,h') mutants while expression in the trunk musculature appears to up-regulated in these mutants. Apostrophes denote alternative views of the same embryo.

The expression patterns of zebrafish *unc45a* and *unc45b* transcripts have been reported, but expression was not examined in the reciprocal *unc45* mutant, which might suggest compensatory regulation between the two homologues [13], [14], [21]. As reported in [21], wild type embryos have a diffuse pattern of *unc45a* expression in the brain and pharyngeal arch region (Fig. 2a). At 48 hpf, we detected a similar *unc45a* expression pattern in *unc45b^−/−^*, and *unc45b^−/−^*; *unc45a^−/−^* embryos (Fig. 2c,d). In contrast to wild type, however, *unc45a* transcripts are almost absent in the *unc45a^−/−^* mutants consistent with nonsense-mediated decay (Fig. 2b). Expression of *unc45a* in the *unc45b^−/−^*; *unc45a^−/−^* embryos appeared slightly more variable than in the unc45a single mutants, although none of the genotypes demonstrated an expansion of the *unc45a* expression domain.

We tested whether *unc45b* expression is affected in the *unc45* mutants (Fig. 2e–h'). Transcripts were detected in the extraocular, cranial, cardiac, pectoral fin, and trunk muscles of *unc45a^−/−^* and wild type embryos (Fig. 2e, f). In contrast, expression is either present at low levels or absent in the extraocular, cranial, cardiac, and pectoral fin muscles of the *unc45b^−/−^* and *unc45b^−/−^*; *unc45a^−/−^* mutants (Fig. 2g, h). Expression in the trunk musculature however, is up-regulated in the *unc45b* mutants as compared to wild type siblings consistent with previous reports [13]. Expression levels of *unc45* genes therefore, are diminished in their respective mutants except in the cases of the *unc45b^−/−^* and *unc45b^−/−^*; *unc45a^−/−^* where *unc45b* expression is increased in the trunk musculature. Since no functional protein is made from this allele, and no expansion of the expression domain is seen, it is unlikely that this can have any compensatory effect on the phenotype.

### *unc45a^−/−^* and *unc45b^−/−^* mutants have different *hsp90* expression profiles

**Figure 3:**
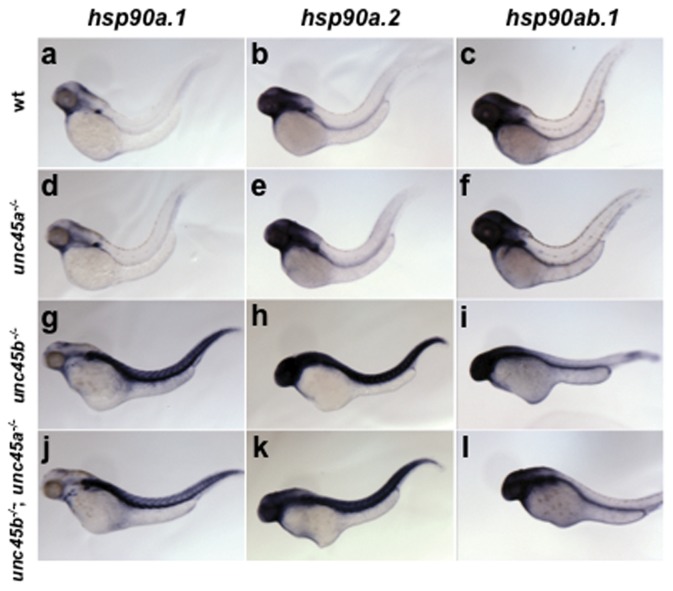
*hsp90a* mRNA is up-regulated in *unc45b^−/−^* mutant embryos at 48 hpf. *In situ* hybridization with anti-sense probes for *hsp90a.1* (a,d,g,j), *hsp90a.2* (b,e,h,k), and *hsp90ab.1* (c,f,i,l) mRNA transcripts was performed on wild type siblings (a–c); *unc45a^−/−^* (d–f); *unc45b^−/−^* (g–i); and *unc45b^−/−^*; *unc45a^−/−^* (j–l) mutants. *unc45b^−/−^* and *unc45b^−/−^*; *unc45a^−/−^* mutants displayed increased expression of *hsp90a.1* (g,j) and *hsp90a.2* (h,k), but not *hsp90ab.1*.

The *hsp90a* chaperone is co-expressed with *unc45b* in striated muscle and the proteins interact *in vitro* [13]. Moreover, the striking phenotypic similarity of the *hsp90a1* and *unc45b* homozygous mutants suggests that this interaction is necessary for myofibrillogenesis [13], [17], [40]. Both *unc45b* and *hsp90a* mRNA transcripts are upregulated in *unc45b^−/−^* mutants; however, expression of the *hsp90* genes has not been examined in the *unc45a^−/−^* mutant [13]. Unc45a (GCUNC45) has been associated with Hsp90 isoforms *in vivo* and *in vitro* [41], [42]. Thus, we examined the expression patterns of *hsp90a.1*, *hsp90a.2*, and *hsp90ab.1* transcripts in *unc45* mutants to determine if a similar relationship exists for *unc45a^−/−^* and *unc45b^−/−^*; *unc45a^−/−^* mutants which could suggest that Unc45a activity affects expression of an Hsp90 chaperone isoform *in vivo*. Whole-mount *in situ* hybridization performed at 48 hpf shows similar expression levels and patterning of the three *hsp90* genes for *unc45a^−/−^* mutants and wild type siblings (Fig. 3a–c, d–f). No appreciable increase in *hsp90* expression levels or spatial distribution was observed in these embryos. A notable increase in *hsp90a.1* and *hsp90a.2* expression was, however, observed in the *unc45b^−/−^* and *unc45b^−/−^*; *unc45a^−/−^* mutants (Fig. 3g, h, j, k). Also, the spatial distribution of *hsp90a* transcripts was expanded in these embryos to include the trunk musculature. In contrast to the *hsp90a* genes, *hsp90ab.1* expression was similar among wild type, *unc45a^−/−^*, *unc45b^−/−^*, and *unc45b^−/−^*; *unc45a^−/−^* embryos (Fig. 3c, f, i, l). Therefore, while this analysis cannot completely rule out a role of Unc45a as an Hsp90 co-chaperone, we can conclude that there is no reciprocal regulatory relationship between *hsp90a* and *unc45a* as is seen with *unc45b*.

### *unc45a* mutants do not show a delay in differentiation of trunk muscle precursors

**Figure 4:**
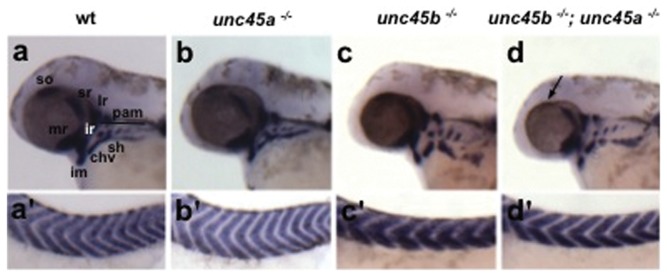
Whole-mount *in situ* hybridization of the myogenic regulatory factor *myoD* in cranial and trunk muscle precursors at 48 hpf. Wild type siblings (a,a'); *unc45a^−/−^* (b,b'); *unc45b^−/−^* (c,c'); and *unc45b^−/−^*; *unc45a^−/−^* (d,d') mutants. Compared to wild type siblings (a) *myoD* expression in the cranial muscle precursors is unchanged in the *unc45a^−/−^* (b), *unc45b^−/−^* (c), and *unc45b^−/−^*; *unc45a^−/−^* (d) mutants. *unc45b^−/−^* (c') and *unc45b^−/−^*; *unc45a^−/−^* (d') mutants have increased expression of *myoD* in the trunk precursors compared to wild type siblings (a') and *unc45a^−/−^* mutants (b'). Primes denote alternative views of the same embryo. chv, constrictor hyoideus ventralis; im, intermandibularis; ir, inferior rectus; lr, lateral rectus; mr, medial rectus; pam, pharyngeal arch muscles; sh, sternohyoideus; so, superior oblique; sr, superior rectus.

Both Unc45a and Unc45b are required during the early stages of myogenesis in mouse C2C12 cells [12]. However, *unc45a* mutants do not display a gross muscle phenotype, nor do we see co-regulation of *unc45a* with *hsp90a*. Given that *myoD* expression is an early marker of muscle commitment, it should reflect any significant delay in myogenic commitment *in vivo*. At 48 hpf cranial myogenesis was normal in *unc45a* mutants as *myoD* expression levels were unchanged compared to wild type siblings in the cranial and extraocular muscle precursors to the constrictor hyoideus, intermandibularis, inferior rectus, lateral rectus, medial rectus, superior rectus, pharyngeal arches, and sternohyoideus (Fig. 4a–d). The *unc45b^−/−^* and *unc45b^−/−^*; *unc45a^−/−^* mutants appear identical, exhibiting a displacement of the intermandibularis, constrictor hyoideus, sternohyoideus, and pharyngeal muscle precursors to positions that are lateral and dorsal to those of wild type siblings (Fig. 4c, d). In the trunk musculature, *unc45b^−/−^* and *unc45b^−/−^*; *unc45a^−/−^* mutants show darker staining *myoD* transcript expression by *in situ* hybridization compared to *unc45a^−/−^* and wild type embryos with levels appearing to be similar between the two (Fig. 4c', d'). However since the somite chevrons are also often compacted in the dorsal/ventral axis in whole mount *unc45b* and other mutants affecting contractility of trunk musculature [13], [43], possibly due to the defect in thick filament contractility, this may not reflect an increase in MyoD expression per cell. As determined by *myoD* expression, the *unc45a^−/−^* mutants display no disruption in myogenesis of the trunk musculature. Moreover, given the observed *myoD* expression in the *unc45b^−/−^*; *unc45a^−/−^* mutants, the lack of *unc45a* in these embryos does not suppress the *myoD* staining seen in the *unc45b^−/−^* mutants, suggesting that *unc45a* does not act epistatically to *unc45b* during zebrafish myoblast differentiation.

### Craniofacial Muscle Organization

**Figure 5:**
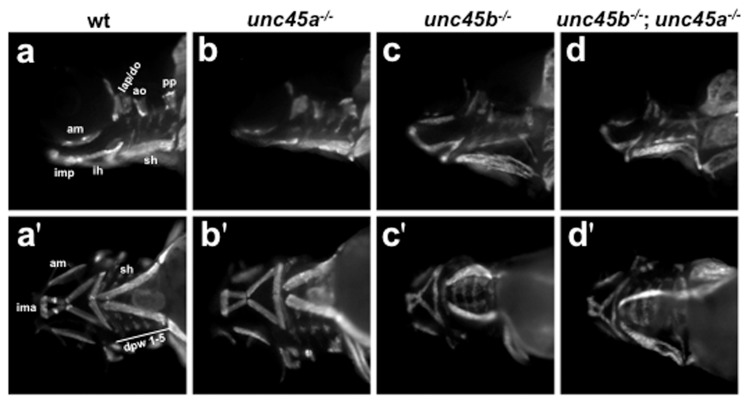
Myosin localization in the craniofacial muscles of 4 dpf embryos. Wild type siblings (a,a'); *unc45a^−/−^* (b,b'); *unc45b^−/−^* (c,c'); *unc45b^−/−^*; *unc45a^−/−^* (d,d') mutants. Lateral (a–d) and ventral (a'–d') views, head to the left. Expression is unaltered between wild type siblings (a,a') and *unc45a^−/−^* (b,b'), *unc45b^−/−^* (c,c') or *unc45b^−/−^*; *unc45a^−/−^* (d,d') mutants. The sternohyoideus is displaced in *unc45b^−/−^* (c,c') and *unc45b^−/−^*; *unc45a^−/−^* (d,d') embryos. Primes denote alternative views of the same embryo. ao, adductor operculi; am, adductor mandibulae; do, dilator operculi; dpw 1–5, dorsal pharyngeal wall; hh, hyohyoideus; ih, interhyoideus; ima, intermandibularis anterior; imp, intermandibularis posterior; lap, levator arcus palatini; pp, pterygoid process; sh, sternohyoideus.

Considering that *unc45b^−/−^* and *unc45b^−/−^*; *unc45a^−/−^* mutants have defective myofibril organization in the trunk musculature, we examined the cranial musculature to see if this muscle population also contained gross morphological abnormalities, a feature that has not been previously described for *unc45* mutants. Although craniofacial myogenesis appeared normal in the *unc45* mutants, we examined the muscle fibre arrangement in older embryos to see if any abnormalities were present (Fig. 5). No cranial muscles were lost in any of the mutants compared to wild type siblings consistent with patterns of *myoD* expression (Fig. 5). The sternohyoideus muscle is displaced laterally in *unc45b^−/−^* and *unc45b^−/−^*; *unc45a^−/−^* mutants and the muscle fibres appear to be bowed in shape. This modified placement of muscle groups is similar to the *myoD* expression observed at 48 hpf and is likely due to the pericardial edema that develops in the *unc45b^−/−^* and *unc45b^−/−^*; *unc45a^−/−^* mutants (Fig. 4).

### Pharyngeal Arch Cartilage is disrupted in *unc45* Mutants

Cranial cartilage and muscle are tissues related by development, function, and evolution. The pharyngeal arches form through the interaction of neural crest, mesoderm, and endoderm tissues, and develop in concert with their associated muscles [36], [44]. Furthermore, both *unc45a* and *unc45b* are expressed in this region: *unc45a* within the arches themselves and *unc45b* in the surrounding musculature [14], [21]. We therefore examined potential *unc45* redundancy in the pharyngeal arches during myoblast differentiation and organization in the *unc45b^−/−^*; *unc45a^−/−^* mutant.

**Figure 6:**
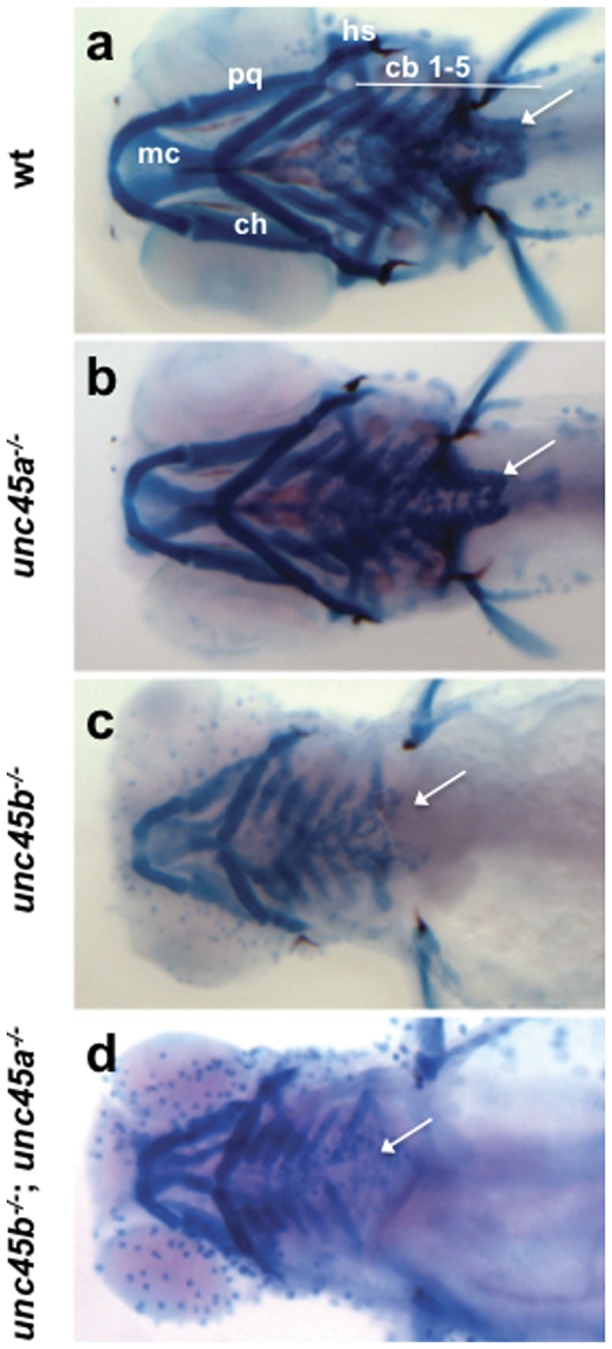
Skeletal defects in *unc45* mutants at 5 dpf. Ventral views of Alcian Blue stained cartilages (a–d). Wild type siblings (a), *unc45a^−/−^* (b), *unc45b^−/−^* (c), and *unc45b^−/−^*; *unc45a^−/−^* (d) mutants. Wild type siblings and *unc45a^−/−^* mutants (a,b) have robust cartilage staining whereas *unc45b^−/−^* (c) and *unc45b^−/−^*; *unc45a^−/−^* (d) mutants exhibit decreased staining, improper angling of the ceratohyal cartilages, and shortening of the palatoquadrates and Meckel's cartilage. The pectoral girdle (arrows) is missing or reduced in *unc45b^−/−^* (c) and *unc45b^−/−^*; *unc45a^−/−^* (d) embryos. cb, ceratobranchial; ch, ceratohyal; hs, hyosymplectic; mc, Meckel's cartilage; pq, palatoquadrate. Small blue dots are an artifact of the fixation and staining process.

The majority of zebrafish skull bones develop indirectly through cartilaginous intermediates that can be visualized using Alcian Blue dye, which stains proteoglycan components of the extracellular matrix of chondrocytes [36]. At 5 dpf, the *unc45b^−/−^* and *unc45b^−/−^*; *unc45a^−/−^* mutants display lower levels of Alcian Blue staining as compared to the *unc45a^−/−^* mutants and wild type siblings (Fig. 6). Reduced staining is seen in all seven pharyngeal arches. Compared to their wild type siblings, the *unc45b^−/−^* and *unc45b^−/−^*; *unc45a^−/−^* mutants (and to a lesser extent the *unc45a^−/−^* mutants) display improper angling of the ceratobranchial and ceratohyal cartilages and shortening of the palatoquadrates and Meckel's cartilage (Fig. 6b, c, d). Also, the pectoral girdle is missing or reduced in the *unc45b^−/−^* and *unc45b^−/−^*; *unc45a^−/−^* mutants and does not connect with the pelvic fins (Fig. 6c, d). Further, *in situ* hybridization of marker gene expression patterns showed no consistent differences between the *unc45b^−/−^* and *unc45b^−/−^*; *unc45a^−/−^* mutants (Figure S2). There is no evidence for patterning defects in pharyngeal neural crest (*hand2, dlx2a*), pharyngeal endodermal pouches (*nkx2.3*), or pharyngeal mesenchyme (*nkx3.2*) (Fig. S2a–d, e–h, i–l, m–p). Moreover, the decreased Alcian Blue staining observed in the *unc45b^−/−^* and *unc45b^−/−^*; *unc45a^−/−^* mutants cannot be associated with defects in the regulation of chondrogenesis as the levels of *sox9a* transcripts remain unchanged in the *unc45a^−/−^*, *unc45b^−/−^*, and *unc45b^−/−^*; *unc45a^−/−^* mutants, compared to wild type (Fig. S2q–t).

## Discussion

Based on reports of at least partial molecular redundancy from several types of *in vitro* assay, it was assumed that the vertebrate paralogs of UNC-45 would have overlapping *in vivo* functions, acting as myosin-specific co-chaperones with primary action at different stages of myogenesis. To directly test this, we undertook a genetic-based examination of the *in vivo* function and potential redundancy between vertebrate *unc45* genes using null alleles of *unc45a* and *unc45b*. Of particular interest for phenotypic examination were regions of the embryo where *unc45* gene expression domains overlap, such as in myoblasts during muscle differentiation and the pharyngeal arches at later stages of development. No phenotype was observed that was novel to the double mutants, nor did the embryos display phenotypes that were more pronounced than those of either of the *unc45a* or *unc45b* mutants alone. This suggests that in contrast to experiments in myoblast cell culture, Unc45a and Unc45b do not participate in redundant functions in the embryo.

We find no evidence for compensation between *unc45a* and *unc45b* or an expansion of *unc45* expression domains in the double mutant. To our knowledge only one other study has examined the effects of both vertebrate *unc45* paralogs. From their work *in vitro*, Price *et al.* proposed that Unc45a and Unc45b play a role in early myogenesis and myoblast fusion [12]. Were this the case *in vivo*, one would expect to see a difference compared to wild type siblings in the extent or timing of expression of the myogenic regulatory factor MyoD in the *unc45a* mutants, which we did not detect. Instead, our findings confirm reports by others that *unc45a* mutants have no discernable muscle phenotypes [21] and show that this is not due to compensation by Unc45b function. Surprisingly, *myoD* expression levels were increased in the trunk muscle precursors of the *unc45b* mutants compared to wild type siblings and *unc45a* mutants. This increase may reflect the similar increase in *hsp90a* levels observed in the *unc45b* mutants. A subset of cells located in the somites and pectoral fin buds express both *myoD* and *hsp90a*, and both genes are down regulated subsequent to the establishment of striated muscle fibres [45], [46]. It was not surprising that *hsp90ab1* expression was similar in all genotypes tested since *hsp90ab1*, unlike *hsp90a*, is not involved in the cellular stress response [17]. In contrast to *unc45b*, the loss of *unc45a* has no effect on the levels of *hsp90* transcripts. This indicates that although both Unc45a and Unc45b can directly interact with Hsp90, and have the ability to act as Hsp90 co-chaperones, they elicit distinct Hsp90 responses at the gene level. This would suggest that *unc45a* and *unc45b* are under differential regulatory control, which may be a reflection of their divergent functions.

Previously, myofibril organization had only been examined in the trunk musculature of *unc45b* mutants. Although craniofacial myogenesis was normal in the *unc45* mutants, we examined the muscle fibre arrangement in older embryos to see if any abnormalities were present. The altered placement of muscle groups in the mutants is similar to the pattern of *myoD* expression observed at 48 hpf and is likely due to the pericardial edema that develops in the *unc45b^−/−^* and *unc45b^−/−^*; *unc45a^−/−^* mutants. The combination of muscle phenotypes observed in the *unc45* mutants indicates that craniofacial myogenesis is unperturbed in the *unc45a* mutants and that the *unc45b^−/−^* and *unc45b^−/−^*; *unc45a^−/−^* mutants have an equal degree of myofibril disorganization. Since no changes in gene expression were observed, it may be that the cartilage defects seen in the *unc45* mutants are associated with pericardial edema. While evidence in support of edema as the origin of a set of universal abnormal pharyngeal arch phenotypes is compelling, it remains to be determined whether the interplay between muscle contraction and cartilage formation is a requirement for proper jaw organization and development. Although the *unc45b^−/−^* and *unc45b^−/−^*; *unc45a^−/−^* mutants accumulate fluid to a much larger extent than the *unc45a^−/−^* mutants, it seems unlikely that edema alone could account for the decreased cartilage staining observed in the *unc45b* mutants. Schilling and Kimmel [36] have proposed that despite the craniofacial muscles differentiating slightly later than cartilages in the same region, the scaffold created through the interaction between the muscle and cartilage precursors might play a role in the patterning of the pharyngeal region.

While the cellular phenotypes of *C. elegans* UNC-45 and fungal UCS protein mutants vary considerably, they all have an effect on myosin assembly and/or function [1], [47]. Consequently, structures that incorporate myosin molecules are also affected in these mutants. Phenotypic differences may be ascribed to the myosin classes with which fungal UCS proteins associate as well as their different domain structure. In contrast to UNC-45, fungal UCS proteins interact with both conventional and non-conventional myosin, leading to their participation in numerous cellular functions [1]. The amino terminal TPR domain is absent in fungi and the central domain, if present, shares little homology with UNC-45 or other fungal proteins [47]. The UCS domain, however, is highly conserved amongst species, with approximately 53% similarity between She4p and human UNC-45 proteins [47].

The abnormal aortic arch development and arterioventricular malformations in the *unc45a^−/−^* mutants remain puzzling. The UCS proteins found in most non-vertebrates function in processes that require non-muscle myosin, such as cytokinesis [22], [48], and data from cell culture studies suggested that Unc45a may have a role in cell division and proliferation, likely mediated by an interaction with non-muscle myosin. We were therefore expecting that the double mutant might exhibit a novel phenotype indicative of cytokinesis defects, but no such phenotype was observed. In an *in vitro* folding assay Liu *et al.* [20] demonstrated that in comparison to Unc45b, Unc45a has a higher affinity for the smooth muscle myosin motor domain and a greater efficiency of folding for this region. Importantly, smooth muscle myosin is closely related to non-muscle myosin II. In human cell lines, Unc45a inhibits retinoic acid signaling and its overexpression in tumors is associated with increased proliferation and metastasis [49], [50]. Unc45a has been linked to cancer progression through its overexpression and ability to confer resistance to histone deacetylase inhibitors and retinoic acid. *In vitro* overexpression of *unc45a* leads to increased cell proliferation and an accumulation of non-muscle myosin and Unc45a at the cleavage furrow during cytokinesis and both proteins also localize to the filopodia of motile cells [49]. In humans, there is a positive correlation between levels of Unc45a and the stage and grade of ovarian cancer. This can be attributed, in part, to the elevated levels of Unc45a protein in ovarian carcinoma tumours compared to healthy ovarian epithelium [49]. This suggests that Unc45a is involved in cellular functions that are not manifested at the embryonic stage we are analysing. Regardless, this analysis has eliminated developmental roles for Unc45a that are dependent on a muscle-myosin-dependent chaperone function, which might have been masked by redundancy with Unc45b.

Since mammals and teleosts have two *unc45* genes, which show no evidence of functional redundancy and appear to be involved in distinct cellular processes, it is useful to consider how these different cellular functions may have arisen. The duplication/degeneration/complementation model (DDC) is based on the observation that many genes required for development have a number of independent functions based on their spatial and temporal expression patterns [51]. One way for both gene duplicates to be preserved is through subfunctionalization, whereby both gene copies accumulate degenerative mutations resulting in the partitioning of the ancestral expression domains and/or functions [52]. When duplicate gene pairs accrue loss-of-function mutations that affect separate sub-functions of the ancestral gene, both gene copies are required to produce the ancestral gene function, and therefore, both copies are retained in the genome [38]. The UNC-45 gene pair was most likely created by a whole genome duplication event some time after the vertebrate lineage branched from that of a common ancestor to *C. elegans* and *D. melanogaster*. Both genes would have been redundant until mutations accumulated following the duplication event. Duplicate gene pairs have a number of potential fates: become a pseudogene, subdivide the ancestral function at the regulatory or protein level (subfunctionalization), develop a new function (neofunctionalization), or a combination of the above. Both vertebrate Unc45b and *C. elegans* UNC-45 are required for myosin motor domain folding and thick filament assembly and are expressed in similar tissues. This suggests that few changes have occurred in the protein domains and regulatory elements responsible for producing the muscle myosin functions of UNC-45 and Unc45b. As the expression pattern and functions of Unc45a appear to have diverged significantly from those of Unc45b in vertebrates, it is likely that expression domain subfunctionalization and neofunctionalization occurred in the Unc45a copy prior to the branching of the bony fish lineage. Had the new Unc45 function not been established after the divergence of lobe and ray-finned fish, either mammals or teleosts, but not both, would have the gene copy encoding a new Unc45 function. The differential distribution of *unc45a* and *unc45b* transcripts suggests that subfunctionalization, which occurred following their duplication, is the likely mechanism for the preservation of Unc45 duplicates in vertebrate genomes.

In conclusion, we have shown that the zygotic-lethal *unc45b^−/−^*; *unc45a^−/−^* and *unc45b^−/−^* mutants are phenotypically indistinguishable both morphologically and at the level of gene expression. No phenotypes were observed in the *unc45b^−/−^*; *unc45a^−/−^* mutant that were novel and/or more severe than any of those found in the single *unc45* mutants as would be expected if *unc45a* and *unc45b* were functionally redundant *in vivo*. Nor did we see any amelioration in the *unc45b^−/−^*; *unc45a^−/−^* embryos compared to *unc45b^−/−^* siblings, suggesting that *unc45a* is not epistatic to *unc45b*. Interestingly, our results highlight major differences between mouse cell culture and zebrafish *unc45a* and *unc45b*. Unlike findings in mouse C2C12 cells, zebrafish lacking *unc45a* display no defects in myocyte function or differentiation suggesting that Unc45a function is not required for myogenesis *in vivo*.

## Supporting Information

Figure S1 **Morphology of 4 dpf** ***unc45*** **mutants.** Wild type siblings (a,a'); *unc45a^−/−^* (b,b'); *unc45b^−/−^* (c,c'); and *unc45b^−/−^*; *unc45a^−/−^* (d,d') mutants. Blood circulates through the hearts of wild type siblings (a,a') and *unc45a^−/−^* mutants (b,b') but not in those of the *unc45b^−/−^* mutants (c,c',d,d'). Cardiac edema is most pronounced in the *unc45b^−/−^* (c,c') and *unc45b^−/−^*; *unc45a^−/−^* (d,d') mutants and absent in wild type siblings (arrowheads). A fully inflated swim bladder is present only in wild type embryos, absent in *unc45b^−/−^* and *unc45b^−/−^*; *unc45a^−/−^* mutants, and minimally inflated in *unc45a^−/−^* mutants (arrows). Somite birefringency is reduced in *unc45b^−/−^* and *unc45b^−/−^*; *unc45a^−/−^* mutants compared to wild type and *unc45a^−/−^* embryos. Apostrophes following a letter denote an increased magnification of the same embryo.
(TIF)

Figure S2 **Pharyngeal arch formation and patterning of** ***unc45*** **mutants is consistent with that of wild type siblings.** Wild type siblings (a,e,i,m,q), *unc45a^−/−^* (b,f,j,n,r), *unc45b^−/−^* (c,g,k,o,s), and *unc45b^−/−^*; *unc45a^−/−^* (d,h,l,p,t) embryos. Expression of: *hand2* at 30 hpf (a–d), *dlx2a* at 30 hpf (e–h), *nkx2.3* at 36 hpf (i–l), *nkx3.2* at 52 hpf (m–p), and *sox9a* at 48 hpf (q–t). Dorsal (a–d, e–h, i–l), ventral (m–p) and lateral (q–t) views. cb, ceratobranchial; p, pharyngeal arch. Numbers denote pharyngeal arch number.
(TIF)

Table S1 **UNC-45 sequences used in phylogenetic analysis.**
(DOC)

## References

[1] Hutagalung AH, Landsverk ML, Price MG, Epstein HF (2002) The UCS family of myosin chaperones. J Cell Science 115: 3983–3990. 12356904 10.1242/jcs.00107

[2] Yu Q, Bernstein SI (2003) UCS proteins: managing the myosin motor. Current Biology 13: R525–527. 12842031 10.1016/s0960-9822(03)00447-0

[3] Venolia L, Ao W, Kim S, Kim C, Pilgrim D (1999) unc-45 gene of Caenorhabditis elegans encodes a muscle-specific tetratricopeptide repeat-containing protein. Cell Motility Cytoskeleton 42: 163–177. 10.1002/(SICI)1097-0169(1999)42:3<163::AID-CM1>3.0.CO;2-E 10098931

[4] Barral JM, Hutagalung AH, Brinker A, Hartl FU, Epstein HF (2002) Role of the myosin assembly protein UNC-45 as a molecular chaperone for myosin. Science 295: 669–671. 11809970 10.1126/science.1066648

[5] Epstein HF, Thomson JN (1974) Temperature-sensitive mutation affecting myofilament assembly in C. elegans. Nature 250: 579–580. 4845659 10.1038/250579a0

[6] Ao W, Pilgrim D (2000) Caenorhabditis elegans UNC-45 is a component of muscle thick filaments and colocalizes with myosin heavy chain B, but not myosin heavy chain A. J Cell Biology 148: 375–384. 10.1083/jcb.148.2.375 PMC2174295 10648570

[7] Venolia L, Waterston RH (1990) The unc-45 gene of Caenorhabditis elegans is an essential muscle- affecting gene with maternal expression. Genetics 126: 345–353. 2245914 10.1093/genetics/126.2.345 PMC1204189

[8] Miller DMd, Ortiz I, Berliner GC, Epstein HF (1983) Differential localization of two myosins within nematode thick filaments. Cell 34: 477–490. 6352051 10.1016/0092-8674(83)90381-1

[9] Kim J, Lowe T, Hoppe T (2008) Protein quality control gets muscle into shape. Trends Cell Biol 18: 264–272. 18495480 10.1016/j.tcb.2008.03.007

[10] Maruyama IN, Miller DM, Brenner S (1989) Myosin heavy chain gene amplification as a suppressor mutation in Caenorhabditis elegans. Mol Gen Genet 219: 113–118. 2575705 10.1007/BF00261165

[11] Hoppe PE, Waterston RH (1996) Hydrophobicity variations along the surface of the coiled-coil rod may mediate striated muscle myosin assembly in Caenorhabditis elegans. J Cell Biol 135: 371–382. 8896595 10.1083/jcb.135.2.371 PMC2121044

[12] Price MG, Landsverk ML, Barral JM, Epstein HF (2002) Two mammalian UNC-45 isoforms are related to distinct cytoskeletal and muscle-specific functions. J Cell Science 115: 4013–4023. 12356907 10.1242/jcs.00108

[13] Etard C, Behra M, Fischer N, Hutcheson D, Geisler R, et al (2007) The UCS factor Steif/Unc-45b interacts with the heat shock protein Hsp90a during myofibrillogenesis. Dev Biol 308: 133–143. 17586488 10.1016/j.ydbio.2007.05.014

[14] Wohlgemuth SL, Crawford BD, Pilgrim DB (2007) The myosin co-chaperone UNC-45 is required for skeletal and cardiac muscle function in zebrafish. Dev Biol 303: 483–492. 17189627 10.1016/j.ydbio.2006.11.027

[15] Janiesch PC, Kim J, Mouysset J, Barikbin R, Lochmuller H, et al (2007) The ubiquitin-selective chaperone CDC-48/p97 links myosin assembly to human myopathy. Nat Cell Biol 9: 379–390. 17369820 10.1038/ncb1554

[16] Walker MG (2001) Pharmaceutical target identification by gene expression analysis. Mini Rev Med Chem 1: 197–205. 12369984 10.2174/1389557013407034

[17] Du SJ, Li H, Bian Y, Zhong Y (2008) Heat-shock protein 90alpha1 is required for organized myofibril assembly in skeletal muscles of zebrafish embryos. Proc Natl Acad Sci U S A 105: 554–559. 18182494 10.1073/pnas.0707330105 PMC2206574

[18] Geach TJ, Zimmerman LB (2010) Paralysis and delayed Z-disc formation in the Xenopus tropicalis unc45b mutant dicky ticker. BMC Dev Biol 10: 75. 20637071 10.1186/1471-213X-10-75 PMC2919470

[19] Bernick EP, Zhang PJ, Du S (2010) Knockdown and overexpression of Unc-45b result in defective myofibril organization in skeletal muscles of zebrafish embryos. BMC Cell Biol 11: 70. 20849610 10.1186/1471-2121-11-70 PMC2954953

[20] Liu L, Srikakulam R, Winkelmann DA (2008) Unc45 activates Hsp90-dependent folding of the myosin motor domain. J Biol Chem 283: 13185–13193. 18326487 10.1074/jbc.M800757200 PMC2442312

[21] Anderson MJ, Pham VN, Vogel AM, Weinstein BM, Roman BL (2008) Loss of unc45a precipitates arteriovenous shunting in the aortic arches. Dev Biol 318: 258–267. 18462713 10.1016/j.ydbio.2008.03.022 PMC2483962

[22] Kachur T, Ao W, Berger J, Pilgrim D (2004) Maternal UNC-45 is involved in cytokinesis and co-localizes with a non-muscle myosin in the early Caenorhabditis elegans embryo. J Cell Science 117: 5313–5321. 15454571 10.1242/jcs.01389

[23] Kachur TM, Audhya A, Pilgrim DB (2008) UNC-45 is required for NMY-2 contractile function in early embryonic polarity establishment and germline cellularization in C. elegans. Dev Biol 314: 287–299. 18190904 10.1016/j.ydbio.2007.11.028

[24] Wong K, Naqvi W, Iino Y, Yamamoto M, Balasubramanian M (2000) Fission yeast Rng3p: a UCS-domain protein that mediates myosin II assembly during cytokinesis. J Cell Science 113: 2421–2432. 10852821 10.1242/jcs.113.13.2421

[25] Westerfield M (2000) The Zebrafish Book. A Guide for the Laboratory Use of Zebrafish (Danio rerio). Eugene, OR. USA: University of Oregon Press.

[26] Kimmel CB, Ballard WW, Kimmel SR, Ullmann B, Schilling TF (1995) Stages of embryonic development of the zebrafish. Developmental dynamics : an official publication of the American Association of Anatomists 203: 253–310. 8589427 10.1002/aja.1002030302

[27] Neff MM, Neff JD, Chory J, Pepper AE (1998) dCAPS, a simple technique for the genetic analysis of single nucleotide polymorphisms: experimental applications in Arabidopsis thaliana genetics. The Plant journal : for cell and molecular biology 14: 387–392. 9628033 10.1046/j.1365-313x.1998.00124.x

[28] Neff MM, Turk E, Kalishman M (2002) Web-based primer design for single nucleotide polymorphism analysis. Trends in genetics : TIG 18: 613–615. 12446140 10.1016/s0168-9525(02)02820-2

[29] Tamura K, Peterson D, Peterson N, Stecher G, Nei M, et al (2011) MEGA5: molecular evolutionary genetics analysis using maximum likelihood, evolutionary distance, and maximum parsimony methods. Molecular biology and evolution 28: 2731–2739. 21546353 10.1093/molbev/msr121 PMC3203626

[30] Larkin MA, Blackshields G, Brown NP, Chenna R, McGettigan PA, et al (2007) Clustal W and Clustal X version 2.0. Bioinformatics 23: 2947–2948. 17846036 10.1093/bioinformatics/btm404

[31] Thisse C, Thisse B (2008) High-resolution in situ hybridization to whole-mount zebrafish embryos. Nature protocols 3: 59–69. 18193022 10.1038/nprot.2007.514

[32] French CR, Erickson T, French DV, Pilgrim DB, Waskiewicz AJ (2009) Gdf6a is required for the initiation of dorsal-ventral retinal patterning and lens development. Dev Biol 333: 37–47. 19545559 10.1016/j.ydbio.2009.06.018

[33] Yelick PC, Schilling TF (2002) Molecular dissection of craniofacial development using zebrafish. Critical reviews in oral biology and medicine : an official publication of the American Association of Oral Biologists 13: 308–322. 10.1177/154411130201300402 12191958

[34] Javidan Y, Schilling TF (2004) Development of cartilage and bone. Methods in Cell Biology 76: 415–436. 15602885 10.1016/s0091-679x(04)76018-5

[35] Walker MB, Kimmel CB (2007) A two-color acid-free cartilage and bone stain for zebrafish larvae. Biotechnic & histochemistry : official publication of the Biological Stain Commission 82: 23–28. 17510811 10.1080/10520290701333558

[36] Schilling TF, Piotrowski T, Grandel H, Brand M, Heisenberg CP, et al (1996) Jaw and branchial arch mutants in zebrafish I: branchial arches. Development 123: 329–344. 9007253 10.1242/dev.123.1.329

[37] Wittbrodt J, Meyer A, Schartl M (1998) More genes in fish? BioEssays 20: 511–515.

[38] Prince VE, Pickett FB (2002) Splitting pairs: the diverging fates of duplicated genes. Nature reviews Genetics 3: 827–837. 10.1038/nrg928 12415313

[39] Volff JN (2005) Genome evolution and biodiversity in teleost fish. Heredity 94: 280–294. 15674378 10.1038/sj.hdy.6800635

[40] Hawkins TA, Haramis AP, Etard C, Prodromou C, Vaughan CK, et al (2008) The ATPase-dependent chaperoning activity of Hsp90a regulates thick filament formation and integration during skeletal muscle myofibrillogenesis. Development 135: 1147–1156. 18256191 10.1242/dev.018150 PMC2358948

[41] Chadli A, Graham JD, Abel MG, Jackson TA, Gordon DF, et al (2006) GCUNC-45 is a novel regulator for the progesterone receptor/hsp90 chaperoning pathway. Molecular and cellular biology 26: 1722–1730. 16478993 10.1128/MCB.26.5.1722-1730.2006 PMC1430258

[42] Chadli A, Felts SJ, Toft DO (2008) GCUNC45 is the first Hsp90 co-chaperone to show alpha/beta isoform specificity. The Journal of biological chemistry 283: 9509–9512. 18285346 10.1074/jbc.C800017200 PMC2442279

[43] Just S, Meder B, Berger IM, Etard C, Trano N, et al (2011) The myosin-interacting protein SMYD1 is essential for sarcomere organization. Journal of Cell Science 124: 3127–3136. 21852424 10.1242/jcs.084772

[44] Hong S-K, Haldin CE, Lawson ND, Weinstein BM, Dawid IB, et al (2005) The zebrafish kohtalo/trap230 gene is required for the development of the brain, neural crest, and pronephric kidney. Proceedings of the National Academy of Sciences of the United States of America 102: 18473–18478. 16344459 10.1073/pnas.0509457102 PMC1311743

[45] Krone PH, Sass JB, Lele Z (1997) Heat shock protein gene expression during embryonic development of the zebrafish. Cellular and molecular life sciences : CMLS 53: 122–129. 9117992 10.1007/PL00000574 PMC11147321

[46] Lele Z, Hartson SD, Martin CC, Whitesell L, Matts RL, et al (1999) Disruption of zebrafish somite development by pharmacologic inhibition of Hsp90. Developmental Biology 210: 56–70. 10364427 10.1006/dbio.1999.9262

[47] Shi H, Blobel G (2010) UNC-45/CRO1/She4p (UCS) protein forms elongated dimer and joins two myosin heads near their actin binding region. Proc Natl Acad Sci U S A 10.1073/pnas.1013038107 PMC3003015 21115842

[48] Lee CF, Melkani GC, Yu Q, Suggs JA, Kronert WA, et al (2011) Drosophila UNC-45 accumulates in embryonic blastoderm and in muscles, and is essential for muscle myosin stability. J Cell Sci 124: 699–705. 21285246 10.1242/jcs.078964 PMC3039016

[49] Bazzaro M, Santillan A, Lin Z, Tang T, Lee MK, et al (2007) Myosin II co-chaperone general cell UNC-45 overexpression is associated with ovarian cancer, rapid proliferation, and motility. The American journal of pathology 171: 1640–1649. 17872978 10.2353/ajpath.2007.070325 PMC2043524

[50] Epping MT, Meijer LAT, Bos JL, Bernards R (2009) UNC45A confers resistance to histone deacetylase inhibitors and retinoic acid. Molecular cancer research : MCR 7: 1861–1870. 19843631 10.1158/1541-7786.MCR-09-0187

[51] Force A, Lynch M, Pickett FB, Amores A, Yan YL, et al (1999) Preservation of duplicate genes by complementary, degenerative mutations. Genetics 151: 1531–1545. 10101175 10.1093/genetics/151.4.1531 PMC1460548

[52] Lynch M, Force A (2000) The probability of duplicate gene preservation by subfunctionalization. Genetics 154: 459–473. 10629003 10.1093/genetics/154.1.459 PMC1460895

