# Supplementary figures and images for: Lack of Developmental Redundancy between Unc45 Proteins in Zebrafish Muscle Development
Source: PLoS One. 2012 Nov 7;7(11):e48861. doi: 10.1371/journal.pone.0048861 (PMC3492250; doi:10.1371/journal.pone.0048861)

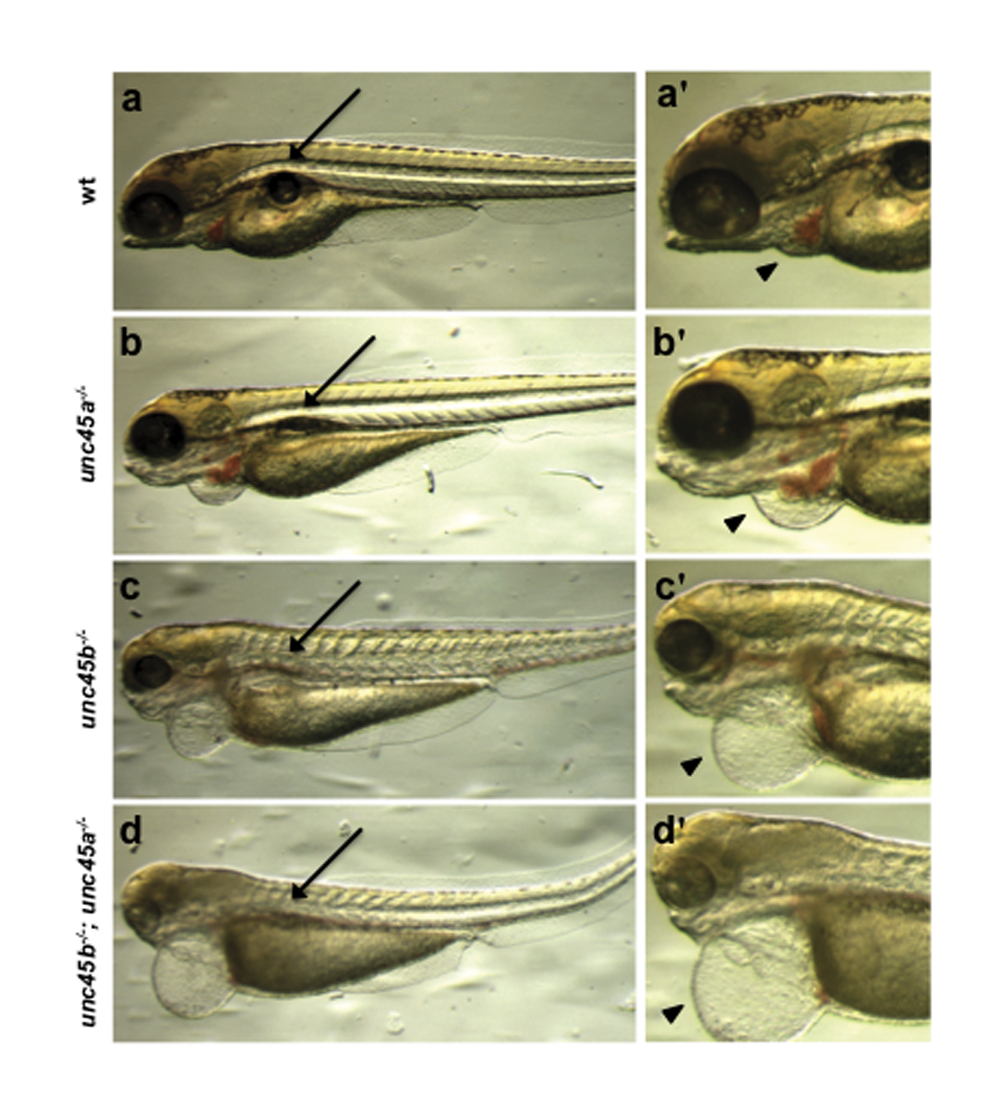

Supplement: Figure S1 — Morphology of 4 dpf unc45 mutants. Wild type siblings (a,a'); unc45a−/− (b,b'); unc45b−/− (c,c'); and unc45b−/−; unc45a−/− (d,d') mutants. Blood circulates through the hearts of wild type siblings (a,a') and unc45a−/− mutants (b,b') but not in those of the unc45b−/− mutants (c,c',d,d'). Cardiac edema is most pronounced in the unc45b−/− (c,c') and unc45b−/−; unc45a−/− (d,d') mutants and absent in wild type siblings (arrowheads). A fully inflated swim bladder is present only in wild type embryos, absent in unc45b−/− and unc45b−/−; unc45a−/− mutants, and minimally inflated in unc45a−/− mutants (arrows). Somite birefringency is reduced in unc45b−/− and unc45b−/−; unc45a−/− mutants compared to wild type and unc45a−/− embryos. Apostrophes following a letter denote an increased magnification of the same embryo. (TIF) [file pone.0048861.s001.tif]

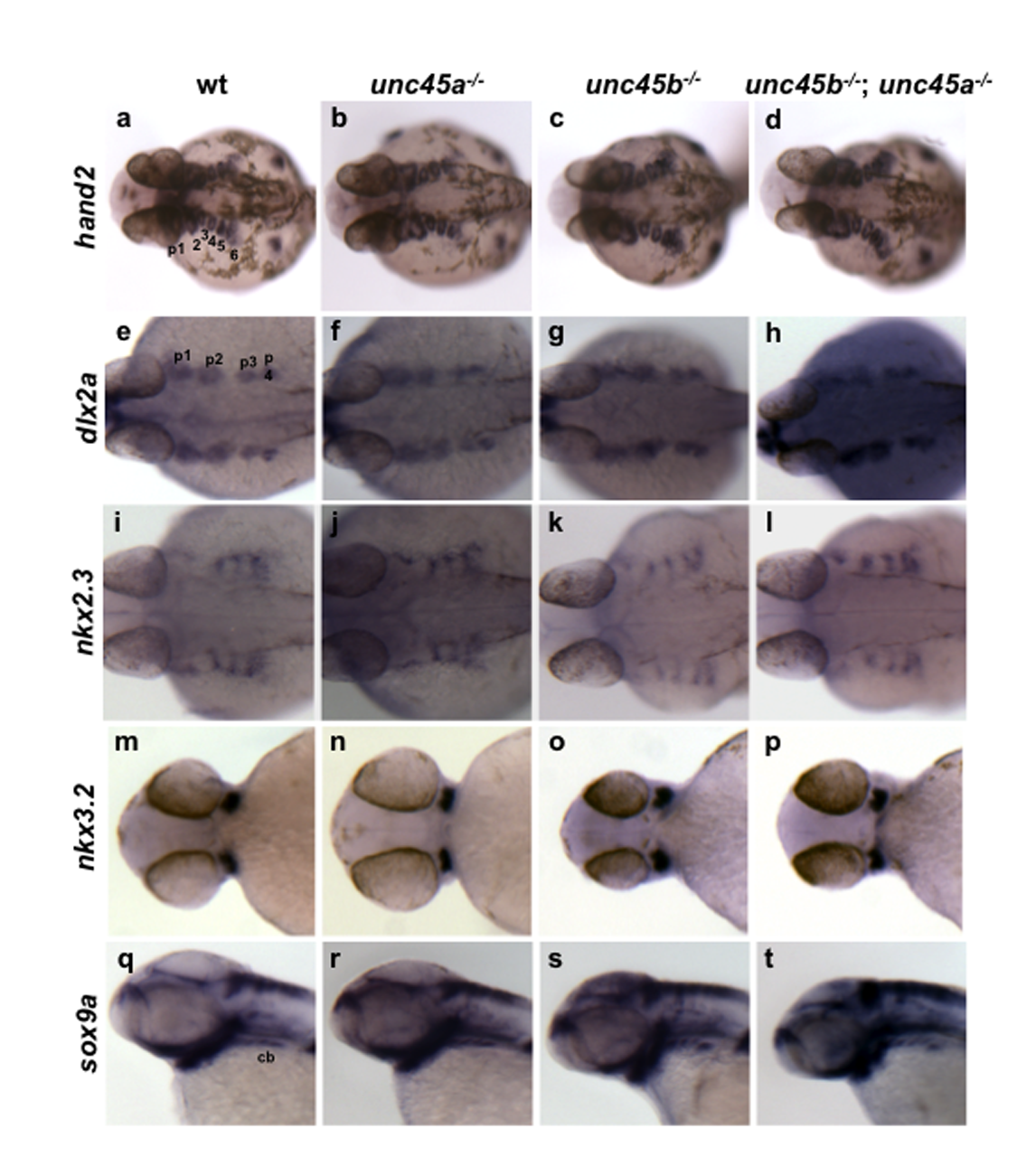

Supplement: Figure S2 — Pharyngeal arch formation and patterning of unc45 mutants is consistent with that of wild type siblings. Wild type siblings (a,e,i,m,q), unc45a−/− (b,f,j,n,r), unc45b−/− (c,g,k,o,s), and unc45b−/−; unc45a−/− (d,h,l,p,t) embryos. Expression of: hand2 at 30 hpf (a–d), dlx2a at 30 hpf (e–h), nkx2.3 at 36 hpf (i–l), nkx3.2 at 52 hpf (m–p), and sox9a at 48 hpf (q–t). Dorsal (a–d, e–h, i–l), ventral (m–p) and lateral (q–t) views. cb, ceratobranchial; p, pharyngeal arch. Numbers denote pharyngeal arch number. (TIF) [file pone.0048861.s002.tif]
