# Supplementary material for: Lack of Developmental Redundancy between Unc45 Proteins in Zebrafish Muscle Development
Source: PLoS One. 2012 Nov 7;7(11):e48861. doi: 10.1371/journal.pone.0048861 (PMC3492250; doi:10.1371/journal.pone.0048861)
Supplement: Table S1 — UNC-45 sequences used in phylogenetic analysis. (DOC) [file pone.0048861.s003.doc]

**Supplemental Table 1** UNC-45 sequences used in phylogenetic analysis.

Abbr. Species UNC-45 Ensembl gene ID Transcript Peptide Genome Location
 Protein Length Length (Chromosome or
 (bp) (aa) Scaffold)

Ce *Caenorhabditis
 elegans* sole F30H5.1 3439 961 Chr. III
Dm *Drosophila
 melanogaster* sole FBgn0010812 3239 947 Chr. 3R
Dr *Danio rerio* a ENSDARG00000003546 3287 935 Chr. 25
 b ENSDARG00000008433 3821 934 Chr. 8
Ga *Gasterosteus
 aculeatus* a ENSGACG00000011505 2820 940 Group XIX
 b ENSGACG00000009985 2802 934 Group XIII
Gg *Gallus gallus* a ENSGALG00000008314 2517 839 Chr. 10
 b ENSGALG00000002186 3305 934 Chr. 19
Hs *Homo sapiens* a ENSG00000140553 4002 929 Chr. 15
 b ENSG00000141161 5675 931 Chr. 17
Mm *Mus musculus* a ENSMUSG00000030533 3635 944 Chr. 7
 b ENSMUSG00000018845 4125 931 Chr. 11
Ol *Oryzias latipes* a ENSORLG00000011861 2814 938 Chr. 6
 b ENSORLG00000009250 3104 930 Chr. 8
Tn *Tetradon
 nigroviridis* a ENSTNIG00000012164 2802 933 Chr. 13
 b ENSTNIG00000015418 2808 935 Chr. 12
Tr *Takifugu
 rubripes* a ENSTRUG00000000546 2808 936 Scaffold 302
 b ENSTRUG00000008123 2814 937 Scaffold 4
Xt *Xenopus
 tropicalis* a ENSXETG00000015452 2745 914 GL172658.1
 b ENSXETG00000013482 3249 927 GL172708.1
